# Supplementary material for: Renal denervation restores biomechanics of carotid arteries in a rat model of hypertension
Source: Sci Rep. 2024 Jan 4;14:495. doi: 10.1038/s41598-023-50816-8 (PMC10767006; doi:10.1038/s41598-023-50816-8)
Supplement: Supplementary file 1 — Supplementary Information. [file 41598_2023_50816_MOESM1_ESM.docx]

**(Supplemental Material)**

# **Renal denervation restores biomechanics of carotid arteries in a rat model of hypertension**

Anastasia Gkousioudi^1^, Margherita Razzoli^2^, Jesse D. Moreira^3^, Gianluca Harbert^2^, Richard D. Wainford^3,4,#^, Yanhang Zhang^1,2,5,#^

^1^Department of Mechanical Engineering, Boston University, Boston, MA, USA

^2^Department of Biomedical Engineering, Boston University, Boston, MA, USA

^3^ Department of Pharmacology & Experimental Therapeutics, Boston University Avedisian and Chobanian School of Medicine, Boston, MA, USA

^4^Division of Cardiology, Emory University School of Medicine, Atlanta, GA, USA

^5^Division of Materials Science & Engineering, Boston University, Boston, MA, USA

^#^Contact author:

| Richard D. Wainford  Division of Cardiology  Emory University  School of Medicine  HSRB II  1750 Haygood Drive  Atlanta, GA 30322  Phone: (404) 727-3754  Email: [rwainfo@emory.edu](mailto:rwainfo@emory.edu) | Yanhang (Katherine) Zhang  Department of Mechanical Engineering  Department of Biomedical Engineering  Division of Materials Science & Engineering  Boston University  110 Cummington Mall  Boston, MA 02215  Phone: (617) 358-4406; Fax: (617) 353-5866  Email: [yanhang@bu.edu](mailto:yanhang@bu.edu) |
| --- | --- |

**
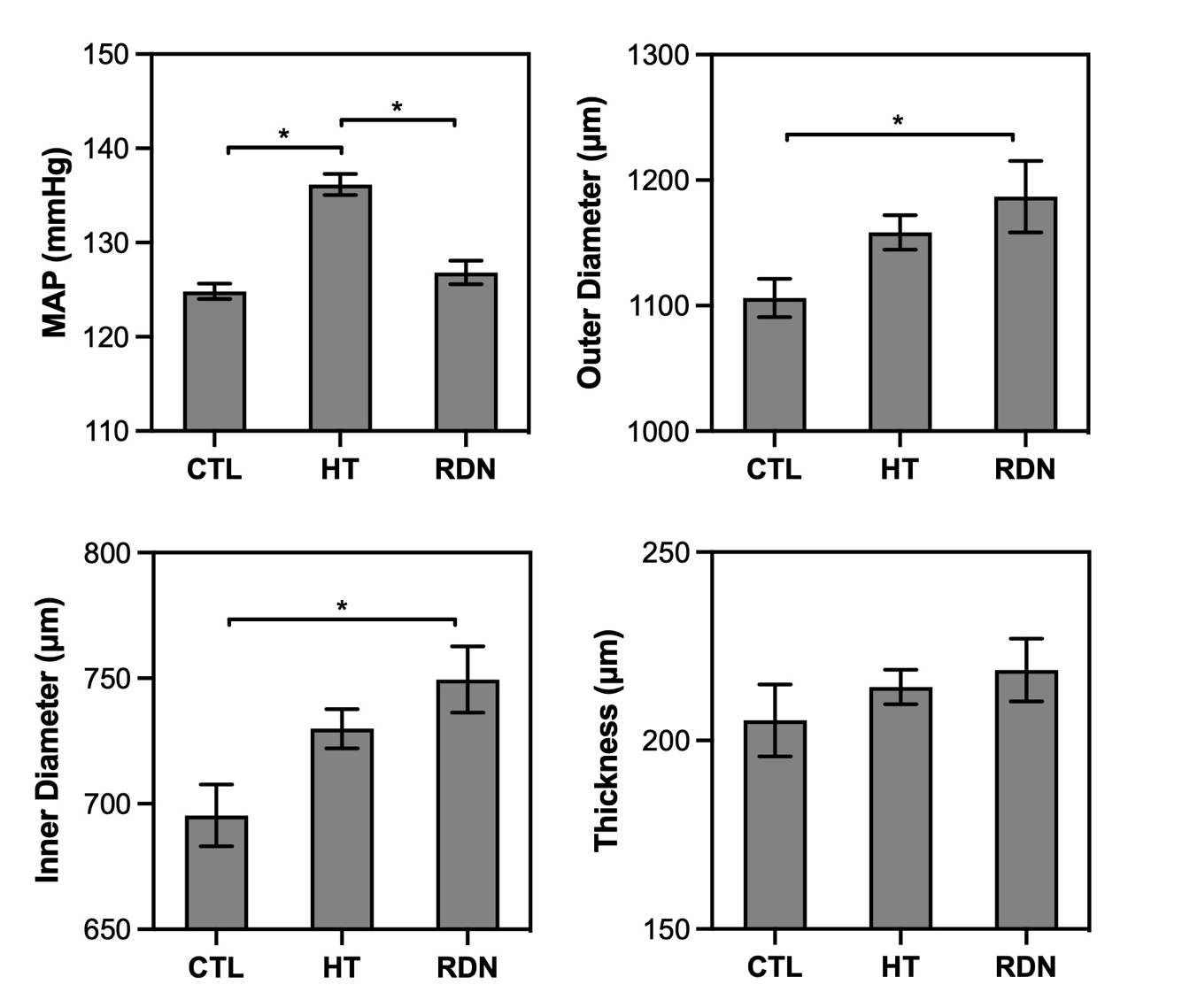
**

**Figure S1.** Average (mean ± SEM) mean arterial pressure (MAP) and arterial dimensions in the undeformed configuration. A: MAP, B-D: Outer diameter, inner diameter and thickness for the control (CTL), hypertensive (HT) and denervated (RDN) groups, *p<0.05

**
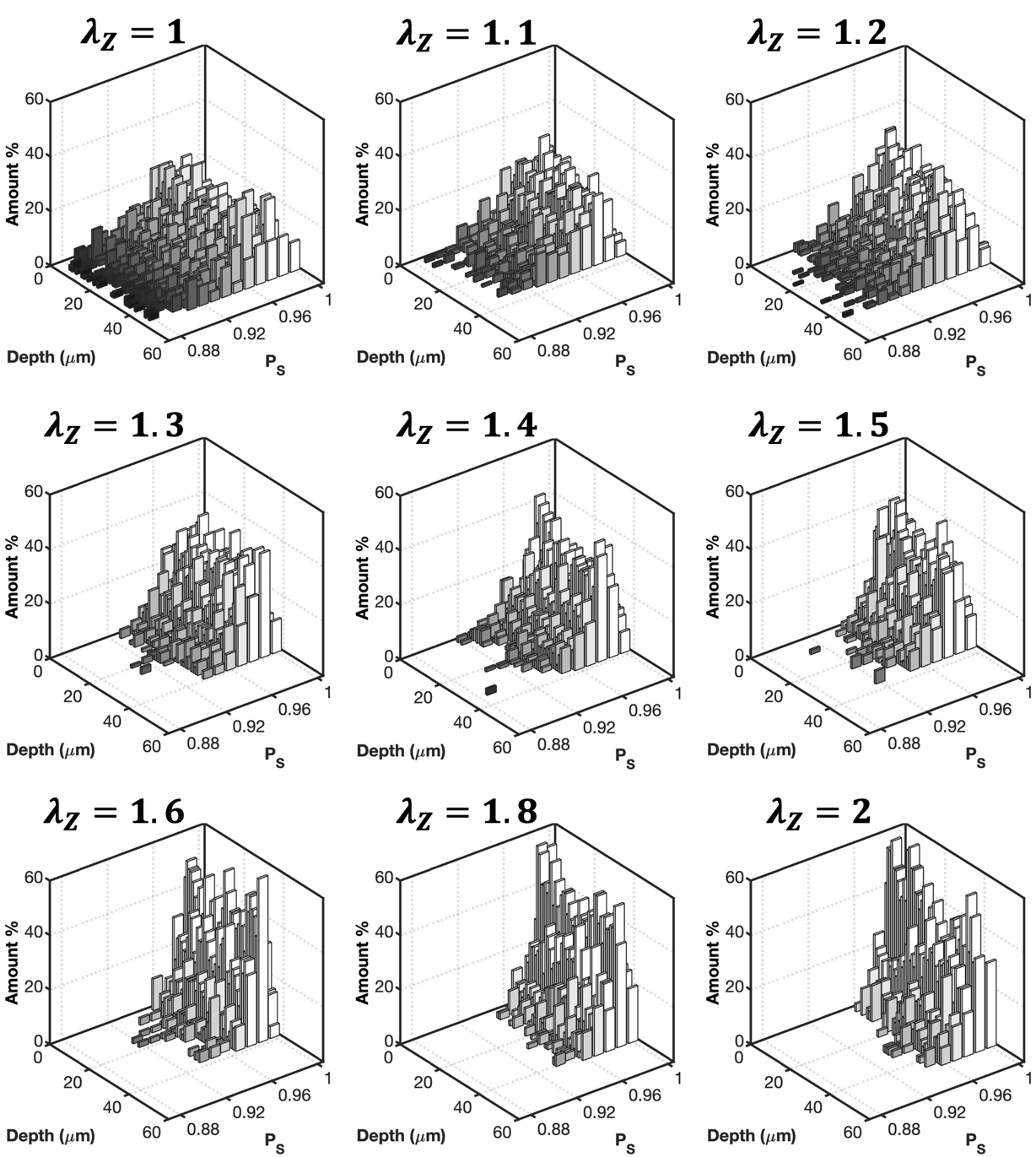
**

**Figure S2.** Representative three-dimensional (3D) histograms of straightness parameter, $P_{S}$ as a function of axial stretching for the hypertensive group. Zero (0 μm) depth corresponds to the outer surface of the adventitia and its value increases up to 50 μm for the middle layers of media.

**
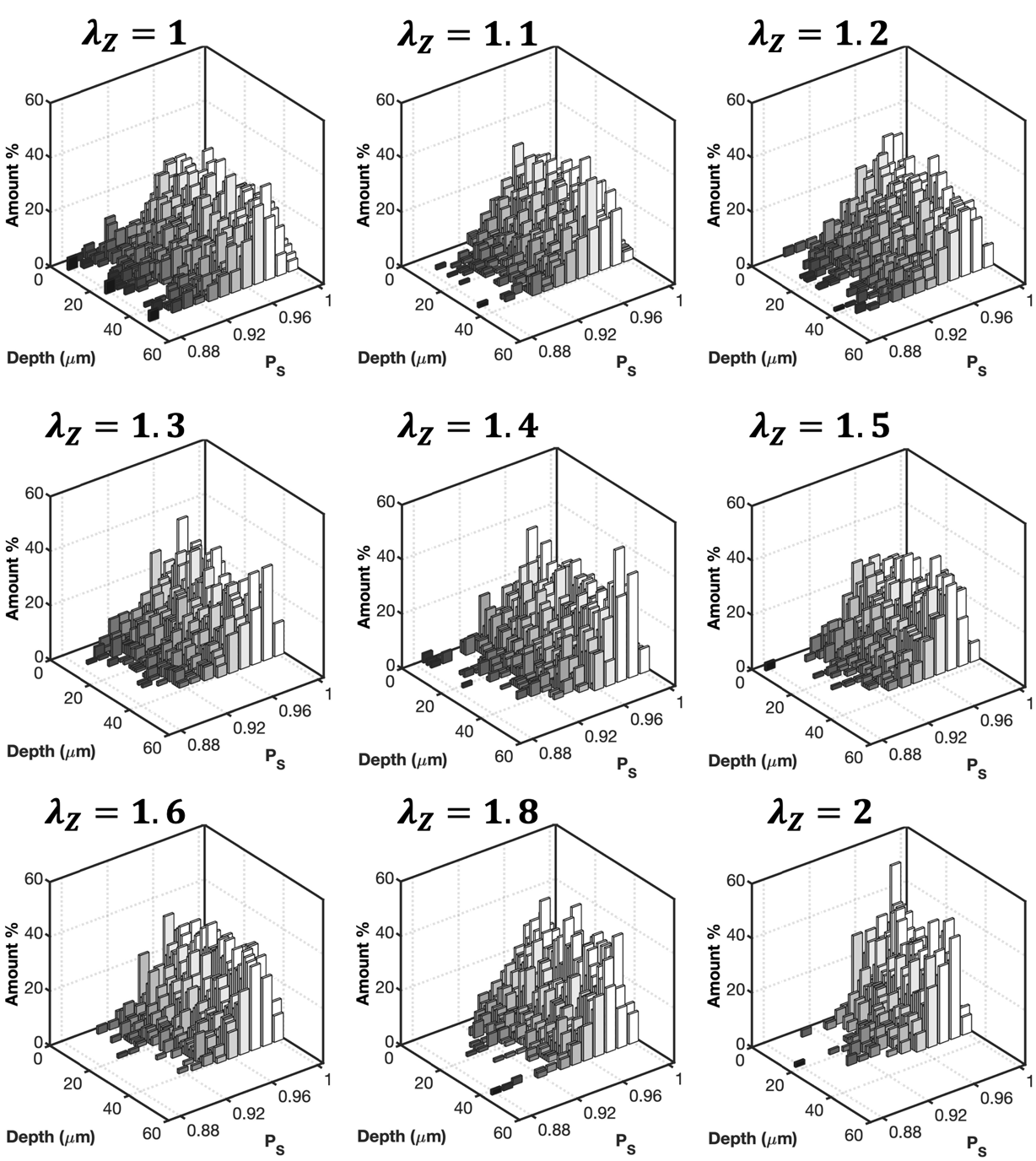
**

**Figure S3.** Representative three-dimensional (3D) histograms of straightness parameter, $P_{S}$ as a function of axial stretching for the denervated group. Zero (0 μm) depth corresponds to the outer surface of the adventitia and its value increases up to 50 μm for the middle layers of media.

**
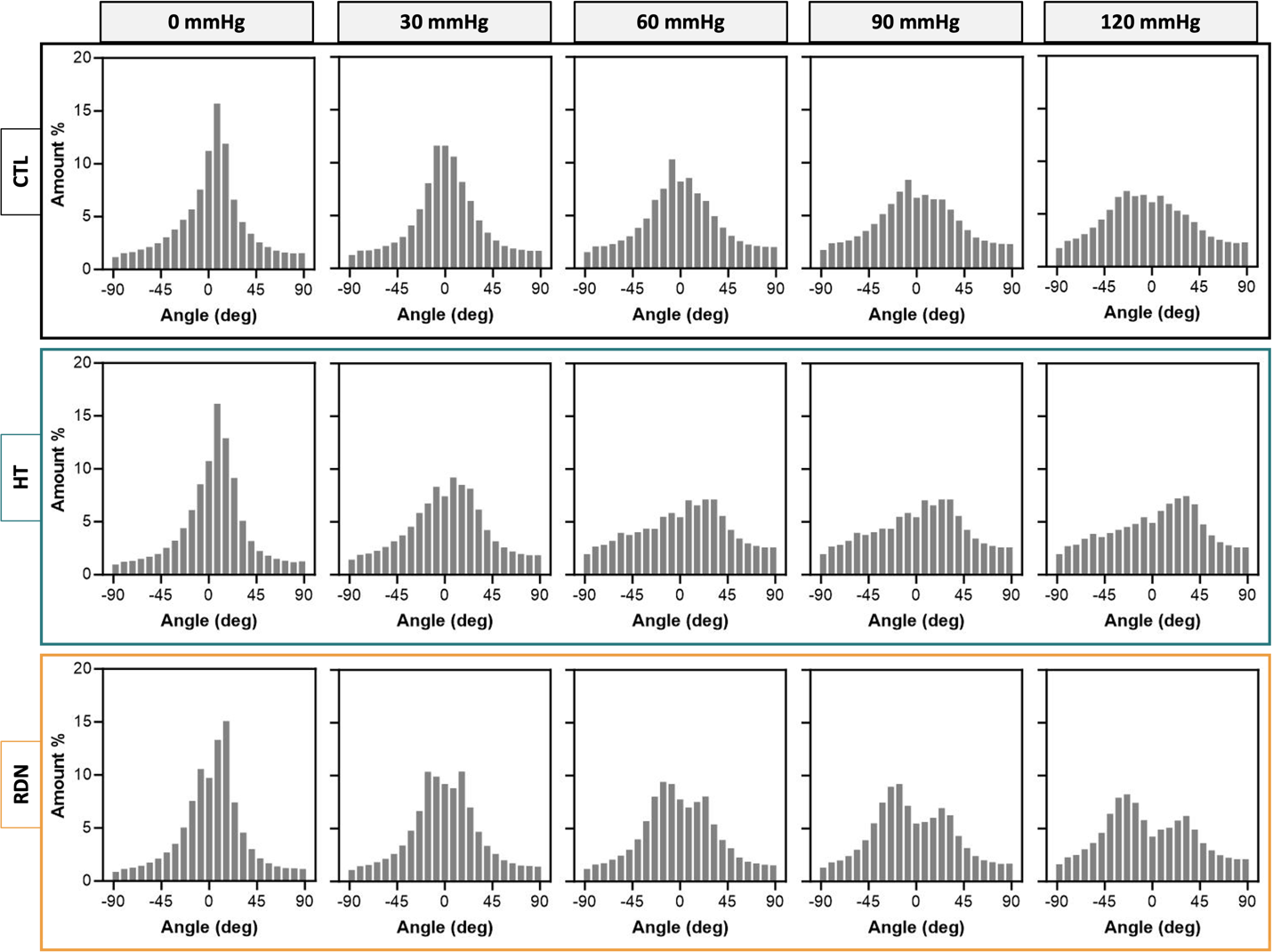
**

**Figure S4.** Histograms of collagen fibers orientation for the control (CTL), hypertensive (HT) and denervated (RDN) group as the pressure increase from 0 to 120 mmHg. Here 0^o^ and ±90^o^ corresponding to the axial and circumferential direction, respectively.

**
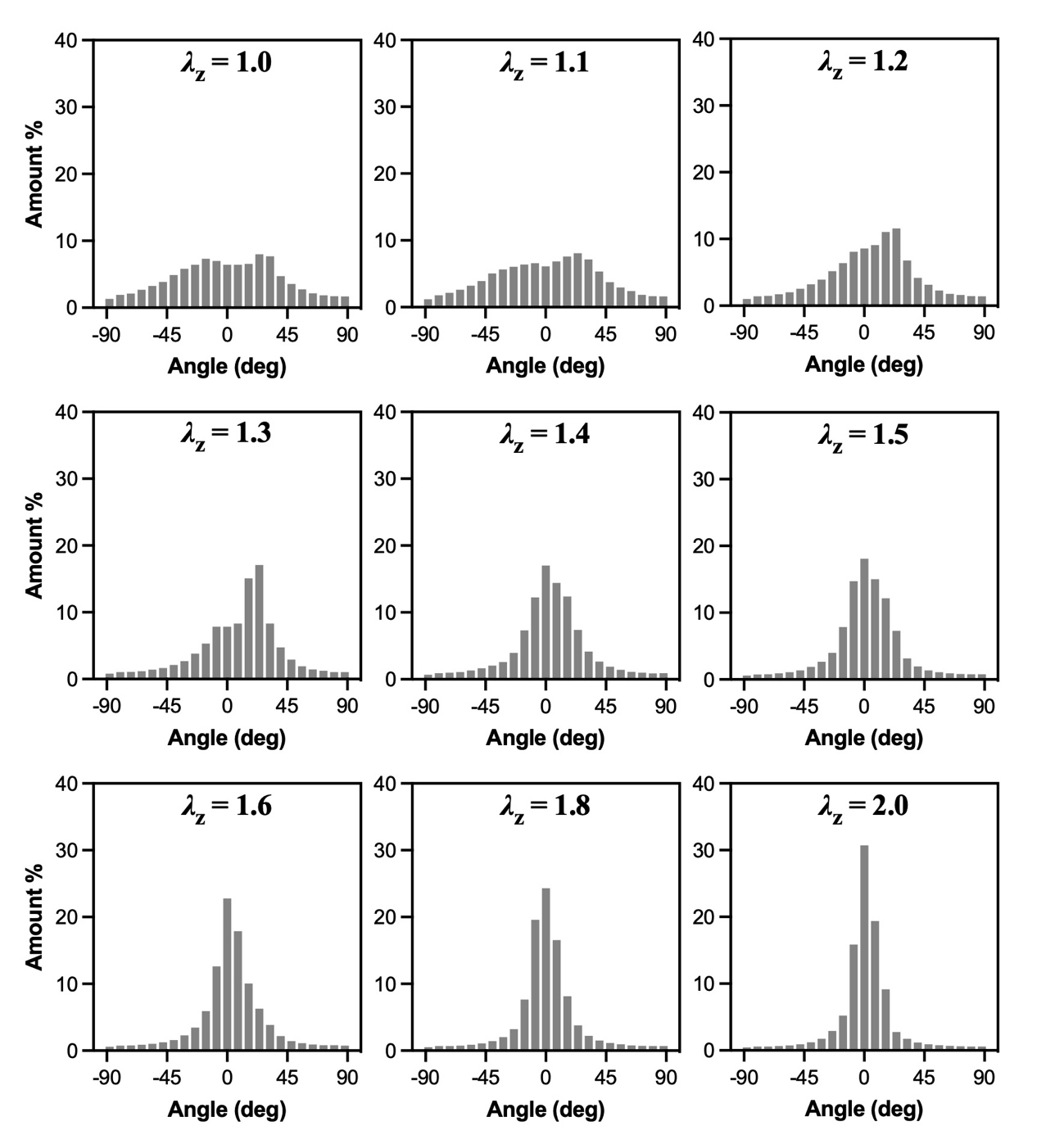
**

**Figure S5**. Histograms of collagen fibers orientation for the control group as a function of axial stretching. Zero degrees (0^o^) correspond to axial direction, while ±90^o^ correspond to circumferential direction.

**
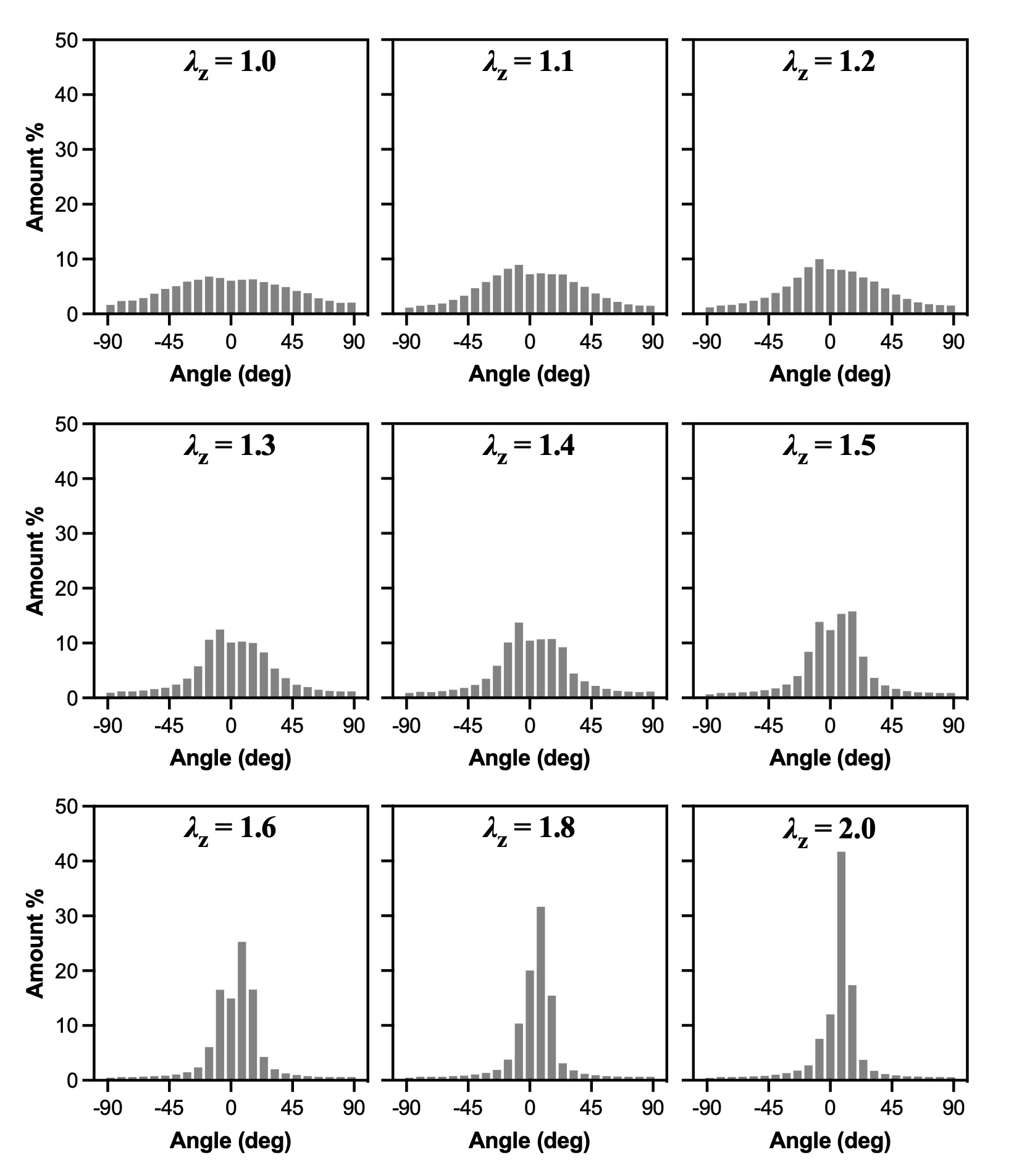
**

**Figure S6.** Histograms of collagen fibers orientation for the hypertensive group as a function of axial stretching. Zero degrees (0^o^) correspond to axial direction, while ±90^o^ correspond to circumferential direction.

**
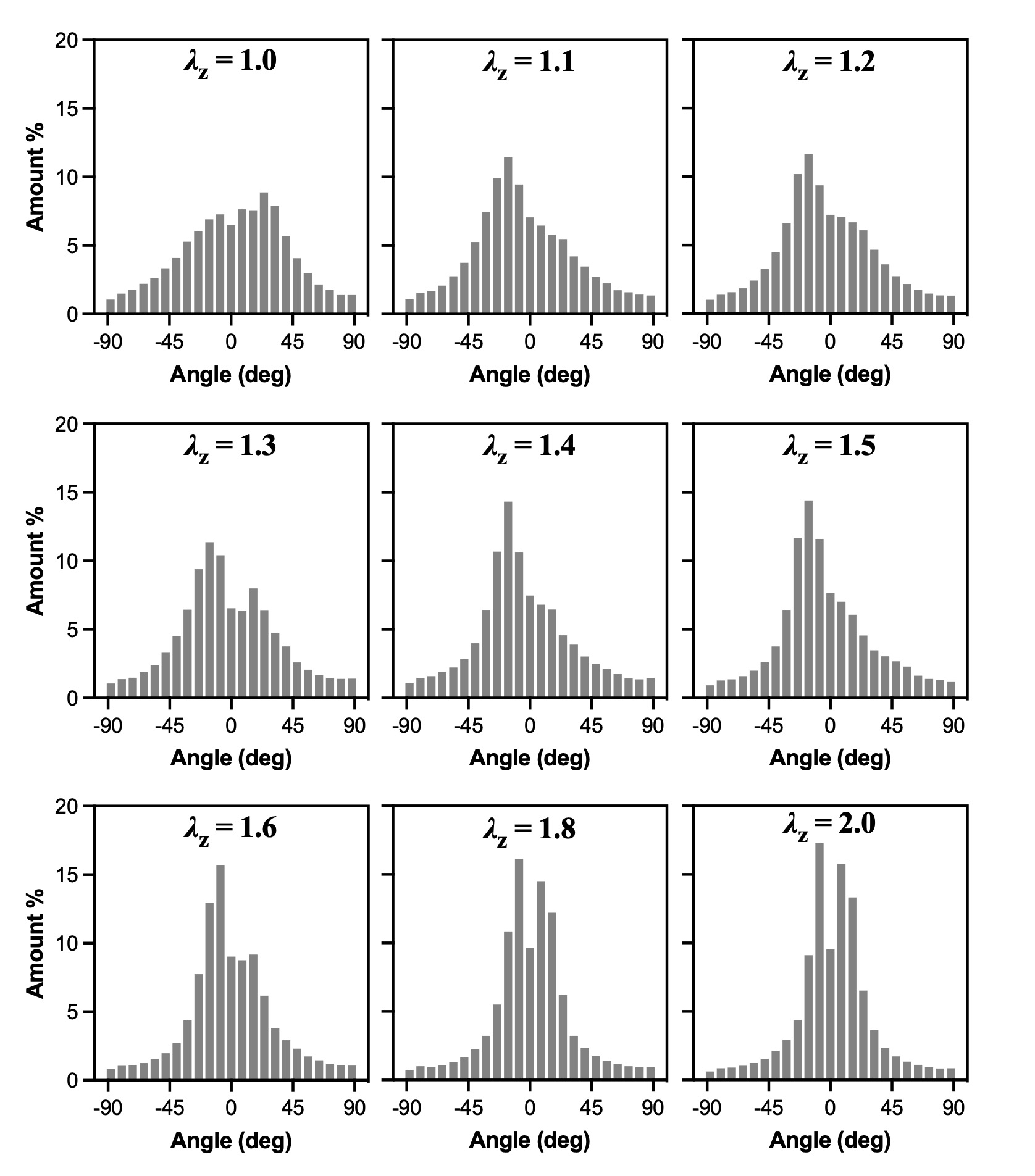
**

**Figure S7.** Histograms of collagen fibers orientation for the denervated group as a function of axial stretching. Zero degrees (0^o^) correspond to axial direction, while ±90^o^ correspond to circumferential direction.
